# The influence of environmental exposure on the response to antimicrobial treatment in pulmonary *Mycobacterial avium* complex disease

**DOI:** 10.1186/1471-2334-14-522

**Published:** 2014-09-29

**Authors:** Yutaka Ito, Toyohiro Hirai, Kohei Fujita, Takeshi Kubo, Koichi Maekawa, Satoshi Ichiyama, Kaori Togashi, Michiaki Mishima

**Affiliations:** Department of Respiratory Medicine, Kyoto, Japan; Diagnostic Imaging and Nuclear Medicine, Kyoto, Japan; Clinical Laboratory Medicine, Kyoto University, Kyoto, Japan; Division of Respiratory Medicine, Takeda General Hospital, Kyoto, Japan

**Keywords:** *Mycobacterium avium* complex, Environmental exposure, Relapse

## Abstract

**Background:**

Environmental exposure is a likely risk factor for the development of pulmonary *Mycobacterium avium* complex (MAC) disease. The influence of environmental exposure on the response to antimicrobial treatment and relapse is unknown.

**Methods:**

We recruited 72 patients with pulmonary MAC disease (male [female], 18 [54]; age, 61.7 ± 10.3 years) who initiated and completed standard three-drug regimens for more than 12 months between January 2007 and December 2011. The factors associated with sputum conversion, relapse and treatment success without relapse were retrospectively evaluated after adjustments for confounding predictors.

**Results:**

Fifty-two patients (72.2%) demonstrated sputum conversion, and 15 patients (28.8%) relapsed. A total of 37 patients (51.4%) demonstrated treatment success. Sputum conversion was associated with negative smears (odds ratio [OR], 3.89; 95% confidence interval [CI], 1.27-12.60; P = 0.02). A relapse occurred in patients with low soil exposure after the start of treatment less frequently than in patients with high soil exposure (7/42 [16.7%] vs. 8/10 [80.0%], P = 0.0003). Treatment success was associated with low soil exposure after the beginning of treatment (OR, 13.46; 95% CI, 3.24-93.43; P = 0.0001) and a negative smear (OR, 2.97; 95% CI, 1.02-9.13; P = 0.047).

**Conclusion:**

Low soil exposure was independently associated with better microbiological outcomes in patients with pulmonary MAC disease after adjusting for confounding clinical, microbiological and radiographic findings.

## Background

Although the prevalence of pulmonary *Mycobacterium avium* complex (MAC) disease has increased in several countries [1–3], the treatment outcomes are unsatisfactory. Even after treatment with clarithromycin- or azithromycin-containing multidrug therapy, a series of pooled studies reported that the sputum conversion rate and the cure rate of pulmonary MAC disease were 68% and 56%, respectively [4].

Various clinical factors, including female gender [5], negative smears [6, 7], and no previous treatment for MAC [6], were reported to be associated with sputum conversion. In the case of a three-times-weekly regimen, a reduction in sputum colony counts was associated with non-cavitary disease, negative smears, no previous treatment for MAC, older age and a longer duration of ethambutol use (for >5 months) [8]. Furthermore, a higher dose of clarithromycin (≥500 mg/day) and the addition of streptomycin to the standard three-drug treatment including clarithromycin, ethambutol and rifamycin were associated with a better microbiological response [7, 9–11].

Relapse after successful treatments with sputum conversion is often observed (8.3-30.0%) [4]. Wallace et al. reported polyclonal infections in human immunodeficiency virus (HIV)-negative patients with pulmonary MAC disease [12] and reinfection by new MAC genotypes after the completion of macrolide therapy [13]. Polyclonal infection could contribute to relapse (reactivation), and the exposure of patients who are susceptible to mycobacterial infection to environmental mycobacteria could also contribute to reinfection (new infection) [13].

Environmental exposure from water and soil in the human living environment is considered the primary route for MAC infection [14]. We reported that patients with pulmonary MAC disease have significantly more soil exposure than non-infected control patients after adjustments for host traits [15] and that approximately one-half of patients’ residential soil contained MAC strains. The clinical and corresponding soil isolates with identical genotypes were identified among patients with a high soil exposure [16]. Furthermore, we recently reported that environmental soil or water exposure was associated with polyclonal MAC infection or mixed mycobacterial infection [17]. These results suggested that environmental exposure is a likely risk factor for the development or reinfection of pulmonary MAC disease. Therefore, we hypothesized that environmental exposures would lead to more MAC infection and influence the responses to antimicrobial treatments and relapse. In this study, we evaluated the significance of environmental exposures on the response to the standard three-drug treatment in patients with pulmonary MAC disease after adjustments for confounding predictors of microbiological responses.

## Methods

### Study population

We recruited 72 patients who met the American Thoracic Society guidelines for the diagnosis of pulmonary MAC disease [18], had newly initiated and completed the standard three-drug treatment (clarithromycin, rifampicin and ethambutol) for more than 12 months between January 2007 and December 2011, these patients and were followed for at least 2 years to define treatment success at the Kyoto University Hospital in Japan. All of the patients received ≥600 mg/day clarithromycin without streptomycin use and either had negative results for serological HIV testing or no obvious risk factors for HIV infection. The institutional review board of Kyoto University approved the research protocol (E1999) and waived the need for obtaining written informed consent for all participants. We obtained written informed consent in the case of patients offering their blood or sputum.

### Data collection

Clinical data, including demographic characteristics, the presence of underlying diseases and conditions and the microbiological results for respiratory specimens, were retrospectively collected. All of the patients completed a questionnaire about their experiences with environmental exposure, such as soil exposure from farming and gardening, or with any activities involving soil exposure, such as digging or carrying soils, turning soil with a spade, mowing grass, planting flowers, and exposure to soil dusts, and water exposure from taking baths or showers, and swimming in a pool [15]; these questionnaires were answered before or at the start of their treatments and during or after discontinuing their treatments. Low soil exposure and high soil exposure were defined as no exposure or less than 2 hours per week and 2 or more hours per week of soil-related activities, respectively [16].

At the start of the treatments or during follow-up, one thoracic radiologist who had no prior knowledge of the patient profiles or the results of the laboratory data conducted chest computed tomography (CT) and/or standard radiographs, which were assessed for the presence of nodules, bronchiectasis and cavities.

Sputum conversion to negative status was defined as three consecutive negative cultures after treatment induction. Sputum relapse was defined as two consecutive positive cultures after sputum conversion [19]. Treatment success and treatment failure included patients who achieved sputum conversion without relapse and patients who did not achieve sputum conversion or relapsed after sputum conversion, respectively.

### Susceptibility to clarithromycin

The minimal inhibitory concentration (MIC) to clarithromycin was determined by the broth microdilution method with commercially manufactured plates (BrothMIC NTM, Kyokuto, Japan). The isolates were assessed as susceptible and resistant to clarithromycin if they had MICs of ≤ 8 μg/ml and ≥32 μg/ml, respectively.

### Statistical analysis

JMP version 10.0 (SAS Institute, Cary, NC, USA) was used for all of the statistical analyses. We divided the patients into two groups; patients with sputum conversion and patients without sputum conversion; patients without relapse and patients with relapse; and patients with treatment success and patients without treatment success. The group comparisons were made using the chi-square test, Fisher’s exact test, and the Wilcoxon test. The comparisons of more than two groups were performed using an analysis of variance. Variables were included in the multiple logistic regression analyses if the probability values were <0.05 according to the univariate analysis. Odds ratios (ORs) and their respective 95% confidence intervals (CIs) were computed as estimates of relative risk. A p-value of <0.05 was considered statistically significant.

## Results

### Characteristics of the pulmonary MAC patients and the microbiological outcomes

The mean patient age was 61.7 ± 10.3 years at the start of treatment, and females predominated (54 patients, 75.0%). Cavities were evident in 35 patients (48.6%), of whom 25 patients (71.4%) were non-smoking females. Among the 15 patients (20.8%) with large cavities (≥2 cm), 12 patients (80.0%) were non-smoking females.

The treatments were discontinued in 61 patients and continued in 11 patients during the observation period. Fifty-two patients (72.2%) converted to negative sputum cultures, and 15 (28.8%) of these patients relapsed. Two patients relapsed within the 12 months of treatment, and 13 patients relapsed after the 12 months of treatment. All 15 strains isolated from each of the 15 relapsed patients were susceptible to clarithromycin. The treatment was successful for 37 (51.4%) patients and failed for 35 patients (48.6%). One patient died during his treatment, and one patient died after discontinuing his treatment. Twenty-five patients experienced high soil exposure during the observation. One patient was working as a farmer, and the remaining 24 of these patients were gardeners. There was no interaction between a negative smear and low soil exposure before the treatments (P > 0.99), after the start of the treatments (P = 0.57) or during the observation (P = 0.89).

### Factors associated with sputum conversion, relapse and treatment success

The patients with sputum conversion had negative smears at the start of the treatment (71.2%) and had either no cavities or cavity sizes of <2 cm (86.5%) (Table 1). The rates of sputum conversion were 84.1% (37/44) in the patients with negative smears versus 53.6% (15/28) in the patients with positive smears (P = 0.005); the sputum conversion rate was 79.0% (45/57) in the patients with no cavities or cavities < 2 cm in size versus 46.7% (7/15) in the patients with cavities ≥2 cm (P = 0.01). In the multiple logistic regression analyses, negative smears were significantly associated with sputum conversion (OR 3.89, 95% CI 1.27-12.60, P = 0.02, Table 2).

**Table 1:** **Characteristics of the patients with pulmonary** ***Mycobacterium avium*** **complex disease with or without sputum conversion**

Variable	Patients with sputum conversion (n = 52)	Patients without sputum conversion (n = 20)	P value
Age, years	61.5 ± 10.2	62.3 ± 10.8	0.85
Gender, female (%)	38 (73.1)	16 (80.0)	0.54
Underlying disease			
Lung disease	12 (23.1)	7 (35.0)	0.30
COPD	3 (5.8)	1 (5.0)	>0.99
Asthma	3 (5.8)	1 (5.0)	>0.99
Previous tuberculosis	2 (3.9)	3 (15.0)	0.13
Previous malignant disease	11 (21.2)	1 (5.0)	0.16
Diabetes mellitus	5 (9.6)	1 (5.0)	>0.99
Collagen vascular disease	6 (11.5)	3 (15.0)	0.70
Immunosuppressive agents	2 (3.9)	3 (15.0)	0.13
Inhaled corticosteroid	2 (3.9)	0 (0.0)	>0.99
Smoking status (ex or current)	11 (21.2)	3 (15.0)	0.74
Alcohol drinking habit	14 (26.9)	3 (15.0)	0.36
Environmental exposure			
Low soil exposure before treatment	39 (75.0)	12 (60.0)	0.21
Low soil exposure during treatment	44 (84.6)	13 (65.0)	0.10
Low soil exposure during observation	37 (71.2)	10 (50.0)	0.09
Bathing (≥1 per day)	50 (96.2)	18 (90.0)	0.31
Shower use in a bathroom	41 (78.9)	14 (70.0)	0.43
Swimming in a pool	2 (3.9)	0 (0.0)	>0.99
Laboratory findings			
Negative smear	37 (71.2)	7 (35.0)	0.005
*M. avium*	39 (75.0)	17 (85.0)	0.60
*M. intracellulare*	12 (23.1)	3 (15.0)	
*M. avium* + *M. intracellulare*	1 (1.9)	0 (0.0)	
Radiographic findings			
Any size of cavity	22 (42.3)	13 (65.0)	0.08
Large cavity (≥2 cm)	7 (13.5)	8 (40.0)	0.02
Bronchiectasis	46 (88.5)	19 (95.0)	0.66
Total length of follow-up time, months	55.6 ± 21.2	49.1 ± 16.4	0.28

COPD, chronic obstructive pulmonary disease. The data show either the numbers (%) of patients or the means ± standard deviations. Low soil exposure is defined as no exposure or less than 2 hours per week of soil-related activities.

**Table 2:** **Factors associated with sputum conversion in patients with pulmonary** ***Mycobacterium avium*** **complex disease**

Variable	Univariate analysis		Multivariate analysis	
	OR (95% CI)	P value	OR (95% CI)	P value
Positive smear	Reference			
Negative smear	4.58 (1.57-14.41)	0.005	3.89 (1.27-12.60)	0.02
Large cavity (≥2 cm)	Reference			
Cavity (none or <2 cm)	4.29 (1.30-14.68)	0.02	3.37 (0.95-12.26)	0.06

OR, odds ratio; CI, confidence interval.

Low soil exposure, particularly after the start of treatment (P = 0.0003), was associated with the absence of a relapse (Table 3). A relapse was observed in only 7 of 42 patients (16.7%) who experienced low soil exposure after the start of treatment, whereas it was observed in 8 of 10 patients (80.0%) with high soil exposure (OR 0.05, 95% CI 0.007-0.25, P = 0.0001).

**Table 3:** **Characteristics of patients with pulmonary** ***Mycobacterium avium*** **complex disease with or without a relapse**

Variable	Patients without relapse	Patients with relapse	P value
	(n = 37)	(n = 15)	
Age, years	60.3 ± 11.4	64.6 ± 5.3	0.27
Gender, female (%)	28 (75.7)	10 (66.7)	0.51
Underlying disease			
Lung disease	7 (18.9)	5 (33.3)	0.29
COPD	1 (2.7)	2 (13.3)	0.19
Asthma	2 (5.4)	1 (6.7)	>0.99
Previous tuberculosis	2 (5.4)	0 (0.0)	>0.99
Previous malignant disease	8 (21.6)	3 (20.0)	>0.99
Diabetes mellitus	3 (8.1)	2 (13.3)	0.62
Collagen vascular disease	3 (8.1)	3 (20.0)	0.34
Immunosuppressive agents	1 (2.7)	1 (6.7)	0.50
Inhaled corticosteroid	1 (2.7)	1 (6.7)	0.50
Smoking status (ex or current)	8 (21.6)	3 (20.0)	>0.99
Alcohol drinking habit	8 (21.6)	6 (40.0)	0.19
Environmental exposure			
Low soil exposure before treatment	31 (83.8)	8 (53.3)	0.03
Low soil exposure after the start of treatment	35 (94.6)	7 (46.7)	0.0003
Low soil exposure during observation	31 (83.8)	6 (40.0)	0.005
Bathing (≥1 per day)	35 (94.6)	15 (100)	>0.99
Shower use in a bathroom	29 (78.4)	12 (80.0)	0.90
Swimming in a pool	2 (5.4)	0 (0.0)	>0.99
Laboratory findings			
Negative smear	27 (73.0)	10 (66.7)	0.74
*M. avium*	26 (70.3)	13 (86.7)	0.44
*M. intracellulare*	10 (27.0)	2 (13.3)	
*M. avium* + *M. intracellulare*	1 (2.7)	0 (0.0)	
Radiographic findings			
Any size of cavity	18 (48.7)	4 (26.7)	0.15
Large cavity (≥2 cm)	6 (16.2)	1 (6.7)	0.66
Bronchiectasis	34 (91.9)	12 (80.0)	0.34
Total length of follow-up time, months	53.8 ± 20.8	60.0 ± 22.1	0.28

COPD, chronic obstructive pulmonary disease. The data show either the numbers (%) of patients or the means ± standard deviations. Low soil exposure is defined as no exposure or less than 2 hours per week of soil-related activities.

Treatment success was associated with low soil exposure, particularly after the start of treatment (P = 0.0002), and negative smears at the start of treatment (P = 0.01) (Table 4). The rates of treatment success were 63.6% (35/55) in the patients with low soil exposure after the start of treatment versus 11.8% (2/17) in the patients with high soil exposure; the rate of treatment success was 61.4% (27/44) in the patients with negative smears versus 35.7% (10/28) in the patients with positive smears. In the multiple logistic regression analysis, low soil exposure after the start of treatment (OR 13.46, 95% CI 3.24-93.43, P = 0.0001) and a negative smear (OR 2.97, 95% CI 1.02-9.13, P = 0.047) were significantly associated with treatment success (Table 5).

**Table 4:** **Characteristics of the patients with pulmonary** ***Mycobacterium avium*** **complex disease with or without treatment success**

Variable	Patients with treatment success	Patients with treatment failure	P value
	(n = 37)	(n = 35)	
Age, years	60.3 ± 11.4	63.3 ± 8.8	0.34
Gender, female (%)	28 (75.7)	26 (74.3)	0.89
Underlying disease			
Lung disease	7 (18.9)	12 (34.3)	0.14
COPD	1 (2.7)	3 (8.6)	0.35
Asthma	2 (5.4)	2 (5.7)	>0.99
Previous tuberculosis	2 (5.4)	3 (8.6)	0.67
Previous malignant disease	8 (21.6)	4 (11.4)	0.25
Diabetes mellitus	3 (8.1)	3 (8.6)	>0.99
Collagen vascular disease	3 (8.1)	6 (17.1)	0.30
Use of immunosuppressive agents	1 (2.7)	4 (11.4)	0.19
Use of inhaled corticosteroid	1 (2.7)	1 (2.9)	>0.99
Smoking status (ex or current)	8 (21.6)	6 (17.1)	0.63
Alcohol drinking habit	8 (21.6)	9 (25.7)	0.78
Environmental exposure			
Low soil exposure before treatment	31 (83.8)	20 (57.1)	0.01
Low soil exposure after the start of treatment	35 (94.6)	15 (42.9)	0.0002
Low soil exposure during observation	31 (83.8)	16 (45.7)	0.0007
Bathing (≥1 per day)	35 (94.6)	33 (94.3)	>0.99
Shower use in a bathroom	29 (78.4)	26 (74.3)	0.68
Swimming in a pool	2 (5.4)	0 (0.0)	0.49
Laboratory findings			
Negative smear	27 (73.0)	17 (48.6)	0.03
*M. avium*	26 (70.3)	30 (85.7)	0.23
*M. intracellulare*	10 (27.0)	5 (14.3)	
*M. avium* + *M. intracellulare*	1 (2.7)	0 (0.0)	
Radiographic findings			
Any size of cavity	18 (48.6)	17 (48.6)	>0.99
Large cavity (≥2 cm)	6 (16.2)	9 (25.7)	0.32
Bronchiectasis	34 (91.9)	31 (88.6)	0.71
Total length of follow-up time, months	53.8 ± 20.8	53.8 ± 19.5	0.90

COPD, chronic obstructive pulmonary disease. The data show either the numbers (%) of patients or the means ± standard deviations. Low soil exposure is defined as no or less than 2 hours per week of soil-related activities.

**Table 5:** **Factors associated with treatment success in the patients with pulmonary** ***Mycobacterium avium*** **complex disease**

Variable	Univariate analysis		Multivariate analysis	
	OR (95% CI)	P value	OR (95% CI)	P value
High soil exposure	Reference			
Low soil exposure	13.13 (3.27-88.87)	<0.0001	13.46 (3.24-93.43)	0.0001
Positive smear	Reference			
Negative smear	2.86 (1.09-7.87)	0.03	2.97 (1.02-9.13)	0.047

OR, odds ratio; CI, confidence interval. Low soil exposure is defined as no exposure or less than 2 hours per week after the start of treatment.

Among the 8 patients who discontinued soil exposure after the start of treatment, 5 patients converted to negative sputum cultures, and 1 patient relapsed. Two patients were newly exposed to soil during the treatment, and 2 patients were exposed after discontinuing treatment. Of these 4 patients, 2 patients converted to negative sputum cultures and relapsed. Among the 13 patients who continued soil exposure after the start of treatment, 8 patients converted to negative sputum cultures, and 6 patients relapsed. More patients who discontinued soil exposure had no relapse and treatment success than the patients who continued or newly began exposure (4/5, 80.0% *vs.* 2/10, 20.0%, respectively, P = 0.09; 4/8, 50.0% *vs.* 2/17, 11.8%, respectively, P = 0.06). Although more patients who began their treatments after 2010 discontinued soil exposure than patients who began their treatments before 2009 (6/10, 60.0% *vs.* 2/15, 13.3%, P = 0.03), there were no differences in the characteristics between the 8 patients who discontinued soil exposure and the 17 patients who continued or newly began exposure.

### Microbiological outcomes and their related factors in cavitary disease

Among the 35 patients with cavities, 22 patients (62.9%) converted to negative sputum cultures, and 4 (18.2%) of these patients relapsed. Treatment was successful for 18 (51.4%) patients. More patients with sputum conversion had negative smears at the start of treatments than the patients without conversion (15/22, 68.2% *vs.* 3/13, 23.1%, P = 0.01). More patients without a relapse had low soil exposure after the start of treatment than the patients with a relapse (18/18, 100% *vs.* 2/4, 50.0%, P = 0.03). Treatment success was significantly associated with the absence of lung disease (0/18, 0.0% *vs.* 5/17, 29.4%, P = 0.02). More patients with treatment success had low soil exposure after the start of treatment than the patients without treatment success (18/18, 100% *vs.* 12/17, 70.6%, P = 0.02).

### Microbiological outcomes and their related factors in non-cavitary disease

Among the 37 nodular bronchiectasis patients without cavities, 30 patients (81.1%) converted to negative sputum cultures, and 11 (36.7%) of these patients relapsed. Treatment was successful for 19 (51.4%) patients. No factors were associated with sputum conversion. More patients without a relapse had low soil exposure after the start of treatment than patients with a relapse (17/19, 89.5% *vs.* 5/11, 45.4%, P = 0.03). More patients with treatment success had low soil exposure after the start of treatment than the patients without treatment success (17/19, 89.5% *vs.* 8/17, 44.4%, P = 0.004).

### Microbiological outcomes and their related factors in *M. avium*disease

Among the 56 patients with *M. avium* disease, 39 patients (69.6%) converted to negative sputum cultures, and 13 (33.3%) of these patients relapsed. Treatment was successful for 26 (46.4%) patients. No factors were associated with sputum conversion. More patients without a relapse had low soil exposure after the start of treatment than the patients with a relapse (24/26, 92.3% *vs.* 6/13, 46.2%, P = 0.003). More patients with treatment success had low soil exposure after the start of treatment than did the patients without treatment success (24/26, 92.3% *vs.* 17/30, 56.7%, P = 0.003).

### Microbiological outcomes and their related factors in *M. intracellulare*disease

Among the 15 patients with *M. avium* disease, 12 patients (80.0%) converted to negative sputum cultures, and 2 (16.7%) of these patients relapsed. Treatment was successful for 10 (66.7%) patients. No factors were associated with sputum conversion, relapse or treatment success. More patients without a relapse had low soil exposure after the start of treatment than did the patients with a relapse (10/10, 100% *vs.* 1/2, 50.0%, P = 0.17). More patients with treatment success had low soil exposure after the start of treatment than the patients without treatment success (10/10, 100% *vs.* 3/5, 60.0%, P = 0.10).

## Discussion

In this study, we demonstrated that low environmental soil exposure was an independent predictor of no relapse and treatment success after adjustments for various clinical factors involving unfavorable microbiologic responses. Because these microbiological outcomes were better associated with soil exposure during or after the start of treatment rather than before treatment, the discontinuation of soil exposure is likely to influence treatment outcomes (Tables 3 and Table 4). Behavioral activities regarding environmental exposure should be considered potential confounding factors in microbiological responses in pulmonary MAC disease.

All participants in this study received the standard three-drug treatment for more than 12 months. The rate of initial sputum conversion in this study was 72.2% (52/72), which is comparable to the results from a series of pooled studies with macrolide-containing treatments (mean 68%, 54-84%) [4]. In several studies, a negative smear was reported to be a better predictor of a microbiological response [8], and it was significantly associated with sputum conversion in this study (Tables 1 and 2).

The population in this study predominantly consisted of non-smoking females with nodular bronchiectatic findings. Although approximately half of the patients (35, 48.6%) had cavitary lesions, and 18 (25.0%) patients had large cavitary lesions (≥2 cm) concomitant with nodular bronchiectasis, few patients had typical apical cavitary disease (male smokers with previous tuberculosis or COPD). It was difficult to differentiate between typical apical cavitary disease and nodular bronchiectasis disease in these patients. We included the patients with cavities in the cavitary disease category.

There have been conflicting reports regarding the influence of the presence of cavitary disease on the efficacy of sputum conversion. One study reported that the absence of cavitary disease resulted in reduced colony counts [8], whereas other studies showed no association between non-cavitary disease and sputum conversion [5, 6]. In this study, a large cavity (≥2 cm), but not any other size cavity, was a significantly unfavorable predictor of sputum conversion.

Wallace et al. reported on reinfection by new MAC genotypes in patients with nodular bronchiectasis [12, 13]. Although there was not a statistically significant difference, a greater amount of relapse was observed in non-cavitary disease patients than in cavitary disease patients in this study (Table 3). The microbiological outcomes were associated with soil exposure alone in the non-cavitary disease patients, whereas they were associated with soil exposure as well as clinical and microbiological factors, such as the presence of COPD and positive sputum smears, in the cavitary disease patients.

There were some limitations in our study. First, when we started the enrollment of patients, we did not intervene to prevent environmental exposure. However, we notified the patients of our findings that soil exposure was a risk factor of pulmonary MAC disease after 2010 [15] and suggested the avoidance of soil exposure and the use of masks when they were exposed to soil. Because we administered a questionnaire about their experiences with environmental exposure before or at the start of their treatments, more patients starting their treatments after 2010 discontinued soil exposure than patients before 2009. This difference is likely a potential confounding variable. However, the patient characteristics did not differ between the patients who discontinued and those who continued soil exposure. Second, we did not analyze the genotypes of the MAC strains collected from the relapsed patients. Therefore, we could not discriminate a true relapse (reactivation) from a new infection (reinfection). Third, because of the small sample size, there were wide confidence intervals in the logistic regression analysis. Finally, we did not include adherence to this regimen as a variable for these microbiological outcomes. However, all the patients had regularly visited our clinics and completed this regimen for more than 12 months.

## Conclusions

In conclusion, we demonstrated an independent association between a low environmental soil exposure and better microbiological outcomes, including no relapse and treatment success, in patients with pulmonary MAC disease after adjusting for confounding clinical, microbiological and radiographic findings.
